# Genetic Association Studies of Suicidal Behavior: A Review of the Past 10 Years, Progress, Limitations, and Future Directions

**DOI:** 10.3389/fpsyt.2016.00158

**Published:** 2016-09-23

**Authors:** Bojan Mirkovic, Claudine Laurent, Marc-Antoine Podlipski, Thierry Frebourg, David Cohen, Priscille Gerardin

**Affiliations:** ^1^Department of Child and Adolescent Psychiatry, CHU Charles Nicolle, Rouen, France; ^2^INSERM Unit U1079, Genetics of Cancer and Neurogenetics, University of Rouen, Rouen, France; ^3^Department of Child and Adolescent Psychiatry, Hôpital Pitié-Salpêtrière, Paris, France; ^4^ICM – Brain and Spine Institute, Hôpital Pitié-Salpêtrière – University Pierre and Marie Curie, Paris, France; ^5^Department of Genetics, CHU Charles Nicolle, Rouen, France; ^6^UMR 7222, Institute for Intelligent Systems and Robotics, University Pierre and Marie Curie, Paris, France; ^7^Laboratoire Psy-NCA-EA-4700, University of Rouen, Rouen, France

**Keywords:** association study, genetics of suicide, suicidal behavior, single-nucleotide polymorphism

## Abstract

Suicidal behaviors (SBs), which range from suicidal ideation to suicide attempts and completed suicide, represent a fatal dimension of mental ill-health. The involvement of genetic risk factors in SB is supported by family, twin, and adoption studies. The aim of this paper is to review recent genetic association studies in SBs including (i) case–control studies, (ii) family-based association studies, and (iii) genome-wide association studies (GWAS). Various studies on genetic associations have tended to suggest that a number of genes [e.g., tryptophan hydroxylase, serotonin receptors and transporters, or brain-derived neurotrophic factors (BDNFs)] are linked to SBs, but these findings are not consistently supported by the results obtained. Although the candidate–gene approach is useful, it is hampered by the present state of knowledge concerning the pathophysiology of diseases. Interpretations of GWAS results are mostly hindered by a lack of annotation describing the functions of most variation throughout the genome. Association studies have addressed a wide range of single-nucleotide polymorphisms in numerous genes. We have included 104 such studies, of which 10 are family-based association studies and 11 are GWAS. Numerous meta-analyses of case–control studies have shown significant associations of SB with variants in the serotonin transporter gene (5-HTT or SLC6A4) and the tryptophan hydroxylase 1 gene (TPH1), but others report contradictory results. The gene encoding BDNF and its receptor (NTRK2) are also promising candidates. Only two of the GWAS showed any significant associations. Several pathways are mentioned in an attempt to understand the lack of reproducibility and the disappointing results. Consequently, we review and discuss here the following aspects: (i) sample characteristics and confounding factors; (ii) statistical limits; (iii) gene–gene interactions; (iv) gene, environment, and by time interactions; and (v) technological and theoretical limits.

## Introduction

There are roughly one million suicides worldwide annually, corresponding to an estimated yearly mortality rate of 14.5 deaths per 100,000 population (1). In Europe, suicide represents the second leading cause of mortality in the 14–24 age groups (2). Suicide constitutes a multifactorial public health issue that involves numerous biological, psychological, cultural, social, and family determinants (3, 4). Support for the implication of genetic risk factors in suicidal behavior (SB) is provided by studies of families (5), twins (6–8), and adoption cases. Studies of adoption have also shown that there is a higher risk of suicide for the individuals who are biologically related to suicidal probands, but not for non-biologically related members of adoptive families (9–11). The recent findings of a large body of studies suggest significant heritability (h2) of completed suicide, with an aggregate estimate of h2 = 45% (3, 12, 13). The heritability appears to depend in part on psychiatric disorders such as mood disorders and substance abuse, with ~90% of suicide attempters having a psychiatric disorder (14–16), and, importantly, to also be partly independent of them (5, 10). This independent factor has been hypothesized to influence impulsive aggression, with individuals who have both of these personality traits and a major mental disorder having the greatest risk of SB (17, 18).

Environmental factors such as early adverse experiences, including sexual and physical abuse during childhood, also strongly impact the risk of SB (19, 20). Some of them are liable to produce direct effects, while others will be controlled through risk for psychiatric disorders, which increases the risk for SB (21). Understanding of the precise genetic system that causes vulnerability to suicidal tendencies is largely incomplete, and efforts to identify the precise molecular mechanisms that are involved have been hampered by the large heterogeneity that is found within groups of SB. The generally accepted and regarded model for the genetic determinism of the SB is a polygenic model that involves a large number of genetic variants, each of which contributes a small modulation of risk. Therefore, association studies that are capable of detecting small effects are likely to be more useful. The majority of studies on genetic SB are based on the hypothesis of “common-disease common-variant.” It is estimated that in the genome, there are more than 10 million common variations (≥5% frequency), most of which are variations of a single base, i.e., single-nucleotide polymorphisms (SNPs).

Two methods are used in particular: genome-wide association studies (GWAS) and gene–candidate association studies. The methodology that has been predominant in the published genetic studies of SB is that of functional candidate–gene studies (with physiopathological hypotheses) (22–26). This review of the literature aims to summarize the results of SB association studies which are currently available. We have also listed the studies of adolescent populations because, to the best of our knowledge, there are no specific reviews of this population. In the second part, we will discuss the limitations of the association studies and new perspectives on the understanding of SB and a broader view of complex diseases.

## Methods

### Literature Search

An electronic search of the literature was performed to identify association studies that investigated the link between genetic variants and SB. A systematic search was conducted using PubMed, SCOPUS, and ISI Web of Science. The key words used to conduct the search were: “suicid*” in association with “gene*,” “polymorphism,” “haplotype,” “association,” “linkage,” or “genome wide.” We also examined the reference sections from the selected papers to identify any additional relevant studies.

### Study Selection

**Figure 1:**
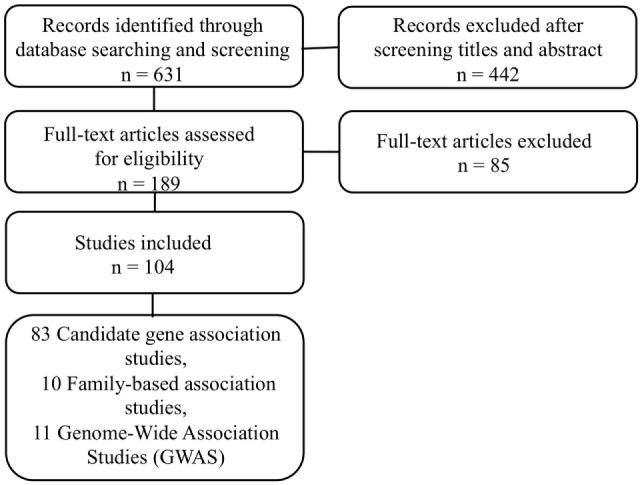
**Flow chart illustrating the selection of suicide behaviors association genetic studies**.

Papers were included in the systematic review if they fulfilled the following criteria: (i) they were published in an English-language peer-reviewed journal from January 2004 to September 2015; (ii) they analyzed the association between any genetic SNP and SB, suicide attempt, or suicide completion; and (iii) they involved adolescent and/or adult subjects. An exclusion of identified irrelevant studies was performed in several steps: (i) duplicates were automatically identified; (ii) studies that referred to non-human subjects were automatically identified; (iii) studies that pertained to cancer research were automatically identified (using keywords such as tumour*, oncolog*, metastas*, or cancer); and (iv) studies of enrolled patients with self-harm without intent to die were identified. The texts of the studies that passed the initial screening were reviewed in extenso and potentially excluded, based on the same criteria. We clustered the retained papers into “candidate–gene association studies” and “genome-wide association studies.” With respect to the PRISMA statement (27), the literature search strategy is summarized in the flow chart presented in Figure 1.

## Results

### Candidate–Gene Association Study

#### Serotonergic System

The serotonergic pathway has been implicated for several decades as having a major role in the pathophysiology of SB (28–30). A number of reviews of the literature, including those of Anguelova et al. (31), Brezo et al. (32), and Antypa et al. (33), have studied the association of SB with common serotonergic gene polymorphisms. These studies mainly pertain to the following: (i) tryptophan hydroxylase (TPH), (ii) serotonin transporter (5-HT), and (iii) serotonin receptors (from 5-HT1 to 5-HT7 with several subtypes and isoforms 5-HT1A, 5-HT2A, and others). We have included 40 studies and 8 meta-analyses. Tables 1 and 2 summarize the studies we selected on the main serotonergic genes.

**Table 1:** **Details of studies included in the review for serotonergic genes**.

Reference	Variants	Suicide outcome/diagnosis	Sample investigated	Findings	Populations
**Tryptophan hydroxylase (TPH1)**					
Zalsman et al. (34)	A218C	SA-adolescent inpatients	88 SA+, and 49 family trios	No association	Jewish
Courtet et al. (35)	A218C	SA-psychiatric patients	103 SA+ (20 repeaters vs. 56)	No association	Caucasian (France)
Ohtani et al. (36)	A779C, A218C	Completed suicide	134 vs. 325 healthy controls	No association	Japanese
Stefulj et al. (37)	C7065T, A218C	Violent suicide victims	60 vs. 284 healthy controls	No association	Caucasian (Croatia)
Liu et al. (38)	A218C, A779C + 3SNP	SA-psychiatric patients	297 SA+, 329 SA−, 184 healthy controls	Haplotype TCAAA of -7180/-7065/-6526/218/779 * SA, OR = 1.62 (1.17–2.24) *p* = 0.00243	Chinese
Stefulj et al. (39)	A218C	Violent suicide victims	247 vs. 320 controls	CC was increased in older (>65 years) victims (*p* = 0.0126) Caucasian (Croatia)	
Viana et al. (40)	A218C	SB-psychiatric patients	248 SA+ vs. 63 healthy controls	No association	Brazilian
Galfalvy et al. (41)	A218C, A6526G	SA-depressive disorders	160 SA+ vs. 183 SA−	AA of both * SA (*p* < 0.01)	Caucasian, African-American
Brezo et al. (19)	143 SNPs (11 genes)	SA-prospective cohort	1121 (follow-up) 117 SA+	rs10488683 * SA, OR = 1.98 (1.21–3.24) *p* < 0.001	Caucasian (Quebec)
Buttenschøn et al. (42)	5 SNP	Completed suicide	572 vs. 1049 controls	No association	Caucasian (Denmark)
Bellivier et al. (43)	A218C	SB-meta-analysis	9 studies: 861cases, 1485 controls	A allele * SB, OR = 1.62 (1.26–2.07)	Caucasian (France)
Li and He (44)	A779C, A218C, A6526G	SB-meta-analysis	34 studies: 3922 cases, 6700 controls	A allelle * SB, OR = 1.14 (1.1–3) *p* = 0.0496	Caucasian (Europe)
Saetre et al. (45)	A218C, A779C + 3 SNP	SB-meta-analysis	12 studies: 1272 cases, 1727 controls	No association	Caucasian (Europe)
Clayden et al. (46)	A218C	SB-meta-analysis	14 studies: 3479 cases, 5945 controls	A allele * SB, OR = 1.22 (1.05–1.41) *p* = 0.007	Various
**Tryptophan hydroxylase (TPH2)**					
Zill et al. (47)	rs1386494, G19918A	CS-alcohol use disorders	263 vs. 263 healthy controls	G allele * suicide, *p* = 0.0038; *p* corr. = 0.038	Caucasian (Germany)
Zhou et al. (48)	G703T, A473T + 13 SNPs	SA-depressive disorders	377 SA+ vs.1652 controls	yin haplotype 212121 * SB in both populations	Finnish and African-American
Ke et al. (49)	rs7305115	SA-MDD	102 SA+ vs. 123 SA−	A allelle * SA *p* = 0.0067	Chinese
Lopez de Lara et al. (50)	14 SNPs	CS-MDD	114 CS vs. 259 SA−	rs4448731, rs6582071, rs4641527, rs1386497 * suicide (*p* < 0.005)	Canadian
Must et al. (51)	G19918A	Completed suicide	288 vs. 327 healthy controls	No association	Estonian
Mouri et al. (52)	A473T, G19918A, G703T	Completed suicide	234 vs. 260 controls	No association	Japanese
Fudalej et al. (53)	rs1386483	Completed suicide	143 vs. 163 controls	TT * suicide (*p* = 0.02), repeated SA (*p* < 0.0001)	Caucasian (Poland)
Zhang et al. (54)	r7305115	SA-MDD	215 SA+ vs. 215 SA−	AA * SB, OR = 0.33 (0.22–0.99) *p* < 0.001	Chinese
Zupanc et al. (55)	A473T	CS-alcohol use disorders	388 vs. 227 controls	No association	Caucasian (Slovenia)
Stefulj et al. (56)	G703T	Violent suicide victims	291 vs. 280 controls	No association	Caucasian (Croatia)
Musil et al. (57)	rs1386494	TESI-depressive disorder	22 TESI, 117 No TESI SA+, 130 SA−	CT + TT * TESI (*p* = 0.0225)	Caucasian (Germany)
Buttenschøn et al. (42)	G19918A + 4 SNPs	Completed suicide	572 vs. 1049 controls	No association	Caucasian (Denmark)
González-Castro et al. (58)	G703T, A473T, G19918A	SB-meta-analysis	37 studies: 4196 cases, 5990 controls	No association	Various

*CS, completed suicide; MDD, major depressive disorder; SA, suicide attempt; SA+, suicide attempters; SA−, patient without a history of suicide attempt; SB, suicidal behavior; SI, suicide ideations; SNP, single-nucleotide polymorphism; TESI, treatment emergent suicidal ideations*.

*Associated with.

**Table 2:** **Details of studies included in the review for serotonergic genes**.

Reference	Variants	Suicide outcome/diagnosis	Sample investigated	Findings	Populations
**Serotonin receptor 1A (5HTR1A)**					
Wasserman et al. (59)	C1019G (HTR1A)	SA-psychiatric patients	272 nuclear family trios	G-allele*SA in a sub-sample selected for high level of previous traumatic life events *t* (*p* = 0.063)	Ukraine
Videtic et al. (60)	C1019G	Completed suicide	323 vs. 190 controls	No association	Caucasian (Slovenia)
Wrzosek et al. (61)	C1019G	SA-alcohol use disorders	38 SA+, 110 SA−	No association	Caucasian (Poland)
Samadi Rad et al. (62)	C1019G	Completed suicide	191 vs. 218 healthy controls	G allele * suicide (*p* = 0.001)	Iranian
Höfer et al. (63)	C1019G + 4 SNPs	SA-resistant depression	160 SA−, 190 SA+	No association	Caucasian, European
Angles et al. (64)	C1019G	SB-meta-analysis	4 studies: 957 cases, 957 controls	No association	Caucasian and Asian
**Serotonin receptor 1B (5HTR1B)**					
Murphy et al. (65)	G861G + 27 SNP	SA-psychiatric patients	76 SA+, 83 SA−	CG * SA (*p* = 0.047)	Caucasian (Ireland)
Zouk et al. (66)	T261G, A161T, C129T, G861C	Completed suicide	338 vs. 358 controls	T allele of A-161T * Suicide (*p* = 0.05)	Caucasian (Quebec)
Kia-Keating et al. (67)	G861C	SB-meta-analysis	7 studies: 789 cases, 1247 controls	No association	Caucasian and Asian
**Serotonin receptor 2A (5HTR2A)**					
Zalsman et al. (68)	T102C	SA-adolescent inpatients	30 nuclear family trios	No association	Jewish Ashkenazi
Giegling et al. (69)	A1438G + 24 SNP	Suicide attempters	203 SA+ vs. 363 healthy controls	Haplotype A–C–T (rs643627-rs594242-rs6311) * SB (*p* = 0.037)	Caucasian (Germany)
Fanous et al. (70)	T102C + 11 SNPs	SI-schizophrenia	722 SI− vs. 127 SI+	No association	Caucasian (Ireland)
Yoon and Kim (71)	A1438G	SA-MDD	181 SA+, 143 SA−, 176 controls	No association	Korean
Wrzosek et al. (61)	T102C	SA-alcohol use disorders	38 SA+, 110 SA−	CC * SA in females (*p* = 0.02)	Caucasian (Poland)
Zalsman et al. (72)	T102C	SA-adolescent inpatients	30 SA+ vs. 95 controls	TT * lower impulsivity (*p* = 0.03)	Jewish
Saiz et al. (73)	A1438G + 7 SNPs	SA-psychiatric patients	227 SA+, 686 SA−, 420 controls	GG * Impulsive SA, OR = 1.88 (1.26–2.83) *p* corr. = 0.016	Caucasian (Spain)
Ben-Efraim et al. (74)	51 SNPs	SA-psychiatric patients	660 nuclear family trios	rs17289304, rs6310, rs6305 * SA	Ukraine
Höfer et al. (63)	A1438G + 4 SNPs	SA-resistant depression	190 SA+, 160 SA−	No association	Caucasian, European
Li et al. (75)	T102C	SB-meta-analysis	25 studies: 1954 cases, 2860 controls	No association	European and Asian
Li et al. (75)	A1438G	SA-meta-analysis	7 studies	No association	Asian
**Serotonin transporter (SLC6A4)**					
De Luca et al. (76)	5-HTTLPR, VNTR	SA-bipolar disorders	86 SA+ vs. 250 SA−	No association	Canadian
Segal et al. (77)	5-HTTLPR	SA-MDD	84 SA+ vs. 152 healthy controls	No association	Brazilian
Wasserman et al. (78)	5-HTTLPR	SA-psychiatric patients	85 SA+	SS * lethality (*p* < 0.0026)	Caucasian, Ukrainian
Segal et al. (79)	5-HTTLPR	SA-MDD	94 SA+ vs. 94 healthy controls	No association	Brazilian
Coventry et al. (80)	5-HTTLPR	SI-community sample	3242 subjects	No association	European (Australia)
Neves et al. (81)	5-HTTLPR	Bipolar disorders	86 SA+, 112 SA−, 103 controls	S allele * violent SA (*p* < 0.0001)	Brazilian
Saiz et al. (73)	5-HTTLPR + 7 SNPs	SA-bipolar disorders	227 SA + , 668 SA−, 420 controls	S allele * lethality, OR = 2.16 (1.15–4.08) *p* = 0.016	Caucasian (Spain)
Hung et al. (82)	5-HTTLPR	SA-schizophrenia	60 SA+, 108 SA−, 302 healthy controls	L allele * SA (*p* = 0.035)	Chinese
Buttenschøn et al. (42)	5-HTTLPR + 4 SNPs	Completed suicide	572 vs. 1049 controls	No association	Caucasian (Denmark)
Lin and Tsai (83)	5-HTTLPR	SA, SC-meta-analysis	18 studies: 1521 SA+ or CS, 2429 controls	No association	Various
Li and He (84)	5-HTTLPR	SA, SC-meta-analysis	39 studies: 3096 cases, 5936 controls	S allele* SA, OR = 0.88 (0.8–0.97) *p* = 0.0068	European, Asian
Clayden et al. (46)	5-HTTLPR	SB-meta-analysis	25 studies: 5363 cases, 9085 controls	S allele * SB, OR = 1.13 (1.05–1.21) *p* = 0.001, I2 = 2.5%	Various

*CS, completed suicide; MDD, major depressive disorder; SA, suicide attempt; SA+, suicide attempters; SA−, patient without a history of suicide attempt; SB, suicidal behavior; SI, suicide ideations; SNP, single-nucleotide polymorphism; TESI, treatment emergent suicidal ideations*.

*Associated with.

##### Tryptophan Hydroxylase

Tryptophan hydroxylase, the rate-limiting enzyme in the biosynthesis of serotonin (5-HT), is a pre-eminent candidate for genetic studies of association in numerous psychiatric disorders, including SB (85). Two genes that code TPH have been identified (TPH1 and TPH2); TPH2 encodes for the main 5-HT-synthesizing enzyme in neurons, while TPH1 is predominantly expressed in peripheral tissue (5, 86).

###### TPH1

Three polymorphisms have been examined extensively: A218C, located in a potential GATA transcription factor-binding site, A779C located on intron 7, and A6526G located on the promoter region. The A allele A218C has been identified to be more frequent in suicide attempters compared to non-attempters (41). Galfalvy et al. (41) showed that the AA genotype on intron 7 and the AA genotype on the promoter were both predictive of attempted suicides during the year-long observation period and were also associated with previous attempts of elevated lethality. This is a prospective study. The methodology used is relatively rare. The study by Courtet et al. (35) also uses a prospective design (1-year follow-up), but they find no association. On a clinical level, it is interesting to note that in the Galfalvy et al. (41) study, intron 7 genotype AA was associated with both fewer reasons for living and greater impulsivity. Regarding the C allele (A218C), only the study by Stefulj et al. (39) shows an association with SB just in people aged over 65 years. However, the association is relatively weak, the number of subjects aged over 65 years is limited (*n* = 74), and the medical records of those who died could not all be obtained.

One study reported an association between haplotype TCAA of the -7180/-7065/-6526/218/779 SNPs and SB and psychiatric disorders (*p* = 0.00243; OR = 1.62; 95% CI 1.17–2.24 and *p* = 0.018; OR = 1.41; 95% CI 1.05–1.91), in the Asian population (38).

The Brezo et al. study (19) is unique in its design because it is a study in a cohort of 1255 members followed longitudinally over 22 years. The subjects were seen at different ages (childhood, mid-adolescence, early adulthood, and mid-adulthood). The DNA was collected at age 27.3 years, on average. The authors took into account environmental factors (family adversity, childhood sexual abuse, and childhood physical abuse) and numerous covariates such as DSM diagnoses, substance abuse, or history of psychopathology. The authors performed separate analyses in the samples of abused subjects and in the total sample. In the univariate analyses, several significant associations were identified in the total sample (TPH1-rs10488683 and HTR2A-rs1885884), and other associations in the sample of victims of sexual abuse (HTR2A-rs1885884, rs7997012, rs6561333 and HTR5A-rs1440449). No gene–gene interaction was found. In the multivariate analyses, only one SNP (TPH1-rs10488683) made a statistically significant contribution independently of gender (OR = 1.2), parental suicide attempts (OR = 2.8), and axis I diagnoses (OR = 2.3). In summary, this study confirms that TPH1 gene variant (rs10488683) is specific to suicide attempts, with its G allele exerting a direct effect that is independent of gender and psychopathology and unmediated by childhood disruptiveness.

We found four meta-analyses that showed significant associations (43, 44, 46). Bellivier et al. (43) included 9 studies among the 15 identified and reported a significant association of the AA218C SNP allele with the risk of suicide in Caucasian populations. The odds ratios associated with the AA genotype and the AC genotype clearly suggest that the A allele increases the risk of SB in a dose-dependent manner, even after removing the two studies that had a significant heterogeneity.

The meta-analysis performed by Li and He (44) was concerned with three polymorphisms (A779C, A218C, and A6526G) and not just A218C. In addition, they included both European and Asian samples and analyzed them both combined and separately. This meta-analysis using multiple methods confirms a strong overall association between SB and the A779C/A218C polymorphisms in both populations. To obtain as much literature coverage as possible, they put equal emphasis on the positive and negative literature to avoid potential publication bias and maximize statistical power and robustness.

Saetre et al. (45) conducted a study with 837 Scandinavian schizophrenia patients and 1473 controls. They showed that three of the five SNPs tested, including A218C and A779C polymorphisms, were associated with schizophrenia susceptibility (*p* = 0.019), but they show no difference in allele frequencies of these loci between affected individuals having attempted suicide at least once and patients with no history of suicide attempts (*p* = 0.84). In the second part of their article, they conducted a meta-analysis of A218C/A779C and SB among individuals affected with a psychiatric disorder. They failed to find any effect of the TPH1 A779C/A218C SNPs on SB (0.96; 95% CI: 0.80–1.16). This result contradicts the hypothesis that TPH1 polymorphisms affect SB independently of mental health status. This is why the authors selected only those studies which had compared allele frequencies in suicidal and non-suicidal patients diagnosed with a psychiatric disorder. Although their study did not take care to distinguish between Asian and Caucasian populations, their results force a more rigorous interpretation of the previously reported association between TPH1 and SB. Thus, one may assume that TPH1 A218C/A779C polymorphisms are associated with increased susceptibility to psychiatric disorders in general, which in turn are characterized by an increased incidence of suicide.

###### TPH2

The human TPH2 gene, situated on chromosome 12q15, consists of 11 exons and covers a region of roughly 93.5 kb. In 2004, Zill’s team was the first to find significant single SNP (rs1386494) and haplotype associations with suicide completion in a German sample (47). In this study, in which TPH2 was linked to SB, 10 single SNPs were used, to define a 28-kb region of the TPH2 gene throughout which LD is elevated, thereby showing that a haplotype block exists anywhere that a responsible functional locus might be found.

We found nine studies that had significant associations in genotypic and/or haplotypic distributions of TPH2 variants between subjects who had SBs and controls (47–50, 53, 54, 57, 87).

For example, the study by Lopez de Lara et al. (50) included subjects who died by suicide during a major depressive disorder (MDD) and controls suffering from MDD. Moreover, all the subjects were well characterized (Axis I and Axis II, psychological autopsy procedure), and bipolar patients were excluded. Four SNPs were identified to be significantly associated with depressed suicide cases and remain significant after statistical adjustments; two SNPs in the TPH2 5′ upstream region (rs4448731 and rs6582071) and two SNPs in introns 1 (rs4641527) and 8 (rs1386497). Alleles T, G, G, and C of SNPs rs4448731, rs6582071, rs4641527, and rs1386497, respectively, were overrepresented in depressed suicide completers. In the second part, the authors conducted a series of logistic regressions to determine a possible interaction between genetic variants and other risk factors, and more specifically whether impulsive–aggressive behaviors (IBAs) may explain the relationship between TPH2 genetic variants and suicide completion. Their results do not confirm this hypothesis.

The variant (rs1386494) was previously identified by Zill et al. (47) but at the same time, the recent study by Buttenschøn et al. (42), which examined over 500 subjects who died by suicide, found no association for TPH2 rs1386494. However, it is important to emphasize the methodology used in this study. The DNA of the cases was obtained from muscle tissue during the autopsy of subjects who died by violent death but with no certainty of suicide. Half of the controls are students for whom there is no clinical information apart from their age and ethnicity. The authors investigated five markers located within four genes (SLC6A4, MAOA, TPH1, and TPH2) involved in the serotonergic system for association with suicide, but they found no robust association.

Zhou et al. (48) showed that in both the tested populations (Finns and African-American), the yin haplotype 212121 was present more frequently in subjects who had attempted suicide. The “risk” haplotype found by Zhou et al. (48) is similar to haplotype 1 of Zill et al. (47), and it was significantly more common among borderline patients (54). Haplotype linkage of TPH2 to SB, major depression, borderline disorder, and cerebrospinal fluid 5-hydroxyindoleacetic acid (a possible mediating phenotype) provides preliminary evidence that there is a functional locus that is potentially within a haplotype block of at least 52 kb in size. In two samples of Chinese depressed patients, Zhang et al. (54) and Ke et al. (49) showed that the TPH2 rs7305115 AA was still a significant protective predictor of SB (OR = 0.33 and OR = 0.35). The findings suggest that the carriers of the A → G mutation of the TPH2 rs7305115 SNP might run a greater risk of attempted suicide than the carriers of the AA homozygous genotype in MDD patients. More particularly, the results suggest that the association between the SNP of the TPH2 gene and tendency to SB in major depression might be distinct from the heritability of mood disorders. Be that as it may, the absence in both studies of potentially functional SNPs indicates a pressing need for investigation of the polymorphisms present in both the TPH2 regulatory and adjacent regions.

Factors that contribute to treatment-emergent suicidal ideation (TESI) using antidepressants have been the focus of recent research strategies. Musil et al. (57) showed that the TPH2 rs1386494 C/T polymorphism continued to display significant association with TESI in comparison with non-TESI (*p* = 0.0018; *p* = 0.0173 after 100,000 permutations). The TPH2 rs1386494 C/T polymorphism had significant predictive power in logistic regression analysis (*p* = 0.0041), displaying an odds ratio of 5.64 (95% CI 1.77–19.58). The haplotype block, which was found in this sample, accords well with the findings of studies conducted by Zill et al. (47) and by Zhou et al. (48). Polymorphisms in the TPH2 gene were studied in previous pharmacogenetic trials on TESI, yet none of these studies found any relevant association (88–91). However, the TPH2 rs1386494 C/T polymorphism was not included in the illumina chip of the STAR*D samples (90, 91) or the recent GWAS (89) (Detailed in the Section “Genome-Wide Association Study”).

##### Serotonin Receptor

###### 5-HTR1A

The 5-HT1A receptor gene (HTR1A) is situated on chromosome 5 (5q11.2-13), and recent studies have found that a common C1019G polymorphism located in its promoter region probably plays a role in depression and SB. Since the first study by Lemonde et al. (92), which reported an association of SB with the rs6295 or C1019G variant, with attempters having a fourfold increased probability of being carriers of the GG genotype, few other teams have found significant associations. The Iranian study by Samadi Rad et al. (62) reported a greater frequency of the G allele in suicide victims compared with control groups. It is worth noting that this team found a higher number of more stressful life events (SLEs) for the subgroup of suicide victims with the GG genotype of C1019G polymorphism. In the same vein, the results obtained by Wasserman et al. (59) indicate a possible role of the G-allele in suicide attempters exposed to high levels of traumatic and/or SLEs. Although interactions with SLEs could be further investigated, the majority of studies reported negative results (59–61, 64, 93).

###### 5-HTR1B

The HTR1B gene is an intronless gene that is located on chromosome 6. The study by Zouk et al. (66) found evidence suggesting a possible role of the promoter variant A161T in suicide. An association was also observed between variation on this locus and levels of IAB. This study, like others, highlighted a possible intermediate phenotype, here IAB levels. IABs may indeed be a mediator, direct or indirect, of the association between genetic factors and suicide. This hypothesis is linked first to clinical observations (impulsivity being a well-identified risk factor) and second to alterations of the serotonergic system (4, 11).

More recently, only the study by Murphy et al. (65) found a significant association between rs6296 and suicide attempts (promoter CpG island of 5-5-HTR1B; *p* = 0.047).

###### 5-HTR2A

The 5-HT2A receptor gene (HTR2A), located on chromosome 13 (13q14–q21), has been implicated in SB. The polymorphisms, which have received the most extensive investigation, are the A1438G (rs6311) and T102C (rs6313). The genetic analyses carried out by Wrzosek et al. (61) found a prevalence of the more common CC genotype in the HTR2A T102C polymorphism in alcoholic subjects who had attempted suicide compared with those who had not made any suicide attempt.

In contrast, no association of T102C with SB was reported in a number of related case–control association studies (70, 71, 95) and one meta-analysis (75). The meta-analysis by Li et al. (75) included a large number of studies, with comprehensive analyses but also analyses in subgroups based on numerous variables such as ethnicity or gender. Although this meta-analysis adopted the random effect model (more conservative than the fixed effects model), significant confusion may arise from the fact that the authors studied two disorders, namely schizophrenia and SB. Moreover, the notion of SB is broad, ranging from suicidal ideation to completed suicide. The authors did, however, detect a significant association between A1438G and SB. Similarly, the study by Saiz et al. (73) showed that the HTR2A A1438A genotype frequencies trended toward being different in impulsive and planned suicide attempts. Genotype A/A was more frequently observed in planned attempts (31.5 vs. 17.9%), whereas the genotype G/G was more common in impulsive attempts (32.9 vs. 15.1%).

Höfer et al. (63) report no association (*p* < 0.05) between any SNP and either risk of suicide or a personal history of attempted suicides. Although interactions between 5HTR2A rs6313 and 5HTR1A rs6295 in risk of suicide and between 5HTR2A rs643627 and 5HTR1A rs6295 in a personal history of attempted suicides have been identified (respectively, *p* = 0.027 and 0.036), the results did not persist after applying correction procedures for multiple testing. The authors conclude that their study fails to find any association of either 5HTR1A or 5HTR2A polymorphisms with a subject’s current risk of suicide or his/her past history of attempted suicide.

Family-based association studies constitute an alternative strategy for studying the variants as against case–control association studies. This approach also has the advantage of reducing the false positive and false negative results in cases of population stratification. Use is generally made of the haplotype relative risk (HRR) method (96). The alleles transmitted to the patient from the parents are compared with the alleles that were not transmitted. The non-transmitted parental alleles serve as controls. In addition to the HRR analyses, the Transmission Disequilibrium Test (TDT) (97) is used to analyze transmitted vs. non-transmitted alleles from heterozygote parents as another indicator of LD. Working with a cohort of 660 nuclear family trios (suicide attempters and both their parents), Ben-Efraim et al. (74) confirms the association between genetic variation (rs6310 and rs6305) in the serotonin 2A receptor (HTR2A) gene and suicide attempt. A large part of this work was devoted to the study of gene–environment (G × E) interaction and included the study of parent-of-origin (POE). This study is of special interest because G × E interactions with SLEs are of importance in a stress–diathesis model of the suicidal process. As regards G × E interactions, the authors report a G × E between the exon 1 SNP rs6313 and exposure to cumulative types of SLEs. This G × E was independent of lifetime physical or sexual assault exposures. The heterozygote risk and TT-homozygote protective effects observed by this team in G × E agree with certain previous genetic findings on SA. In addition, their exploratory analysis revealed a significant POE in this G × E in female subjects, which followed a polar overdominant inheritance pattern. POE in the presence of G × E suggests the importance of non-Mendelian inheritance patterns of HTR2A in the association with SA observed by Ben-Efraim et al. and may perhaps explain some of the inconsistencies in the genetic observations previously reported by others.

##### Serotonin Transporter

The serotonin transporter (5-HTT) plays an important role as a regulator of serotogenic signaling at synapses. The gene of serotonin transport (SLC6A4) is situated on chromosome 17 (17q11.2); it has a common functional promoter polymorphism (5-HTTLPR, rs4795541), which consists of a short (S) and a long allele (L). The L allele of this marker has been found to transcribe the gene two to three times more efficiently than the S allele does (98). This candidate gene has been the subject of extensive study, and the results are divergent. Certain studies have reported an association of the S-allele with suicide (77, 78, 81), whereas other studies reported no significant difference (42, 80). The results of the Clayden et al. (46) meta-analysis of random-effects show that the minor (S) allele in SLC6A4 enhanced the risk of attempted suicide by 13% [OR = 1.13 (1.05–1.21), *p* = 0.001], with heterogeneity being low (I2 = 2,5%). A meta-analysis by Li and He (84) that comprised 39 studies suggests an association of the S-allele of 5-HTTLPR with SB.

When a pairing was made between suicide attempters and non-attempters who had the same psychiatric disorders, the carriers of the long allele (L) were found to be associated with reduced suicide risk (OR = 0.83; 95% CI: 0.73–0.95).

A meta-analysis of the results from 18 studies (83) failed to detect an association of 5-HTTLPR with SB. However, when the comparison only concerned patients diagnosed with the same psychiatric conditions, the frequency of the S-allele was found to be significantly more elevated in subjects having attempted suicide. Furthermore, it must be noted that several authors have reported an association of the S-allele with violent suicide (73, 78, 81, 83).

Hung et al. (82) examined the link between the tri-allelic 5-HTTLPR and SB among Chinese patients with schizophrenia and examined whether the use of violent methods in suicide attempts is influenced by the polymorphism. In their analysis, the authors used the LA-dominant model and found that the LA allele carriers were significantly more likely to have attempted suicide (*p* = 0.035); a comparable association was also reported between the LA allele and suicide attempts by violent means (*p* = 0.028). On the other hand, when the traditional biallelic 5-HTTLPR was investigated, no association was detected. These results are different from those found in Caucasian subjects (79), for whom no associations have been identified.

#### Dopaminergic and Adrenergic Receptors

##### Dopaminergic Receptor

Suda et al. (99) conducted an investigation into two dopaminergic D2 receptor (DRD2) genetic polymorphisms (TaqIA and -141C Ins/Del) in 120 Japanese suicide attempters and 123 volunteers having no connection with them. The authors reported significant disparities between the suicide attempt group and the healthy control group (-141C Ins/Del, *p* = 0.01; TaqIA, *p* = 0.036) as regards the genotype and allele frequencies of polymorphisms. This result confirmed an earlier finding of an association between the DRD2-141C Del variant with suicide attempts in a group of German alcoholics (100). Studies of the AKT1 and AKTIP genes investigated the association of encoding proteins, which are key to the identification of dopamine and serotonin neurotransmitter systems, with SB observed in bipolar subjects. The results obtained showed AKT1 to be associated with cases of attempted suicide (rs1130214) and violent attack attempts (rs2494746) (101), but not AKTIP.

##### Adrenergic Receptor

The ADRA2A gene, composed of an intronless, single 3650-bp exon, is situated on chromosomes 10q24-q26, in other words, a region which has been found to be associated with attempted suicides independently of disease phenotypes (102). A number of genetic variants of the ADRA2A gene, including the promoter genetic variants N251K, have been reported to be associated with a tendency to completed suicide (103). However, Martín-Guerrero et al. (104) failed to find any association of the N251K SNP with suicide completion. Using a case–control association study of 184 completed suicides and 221 control subjects in a Japanese population, Fukutake et al. (105) found that C-1291G SNP in the promoter region was significantly associated with suicide in females (*p* = 0.043 and 0.013 for genotypic and allelic comparisons, respectively).

#### Catabolism of Monoamines

##### Catechol-*O*-Methyltransferase

Catechol-*O*-methyltransferase (COMT) is encoded by a single gene that is localized on chromosome 22q11.1-q11.2 and represents a major enzyme in catecholamine inactivation. A common SNP, Val158Met in exon 4 of the COMT gene, has been observed as being linked to various psychiatric disorders, one of which being suicide (106). This SNP displays a commonly occurring functional polymorphism, a G to A nucleotide transition causing the substitution of amino acid from valine (Val) to methionine (Met) at position 158 COMT Val(108/158) Met (rs4680). This SNP (rs4680) affects the functional ability of the enzyme to catabolize synaptic proteins, such as that in human postmortem PFC tissue; the Val/Val genotype is associated with ~38% greater enzyme activity than that observed in Met/Met homozygotes (107). Various studies have demonstrated associations of the COMT Val158Met gene polymorphism with SB (108, 109), although contradictory results have also been reported (110, 111).

A meta-analysis of six-related studies suggested an association of the COMT Val158Met polymorphism with SB, and this relationship was moderated by gender and the lethality of the suicide attempt (67). However, only two studies have been carried out to investigate the association of the COMT Val158Met polymorphism with completed suicides (108, 109). Pivac et al. (109) found significant disparities in how the COMT Val(108/158) Met variants (genotypes, alleles, and Val carriers) are distributed, but only in males, between suicide victims and controls (*p* = 0.018, *p* = 0.031, *p* = 0.005) and between violent suicide victims and controls (*p* = 0.026, *p* = 0.042, *p* = 0.010). Ono et al. (108) found a significant difference between males having committed suicide and the male control group (*p* = 0.036), whereas occurrence of the high-activity COMT Val/Val genotype was significantly less frequent in males having committed suicide than in the male control group (OR: 0.52; 95% CL: 0.31–0.89; *p* = 0.016).

In contrast, more recent meta-analyses that included new data questioned the association of COMT Val158Met with SB (111, 112) and did not report any association. Calati et al. (112) presented a revised meta-analysis of 12-related studies, with the authors failing to find any association of SB with rs4680 after consideration of both genotypes and the frequency of alleles. Further to that, the authors conducted a number of sensitivity and meta-regression analyses, designed first to consider ethnically homogeneous groups; second, to compare suicide attempters vs. non-attempters in a cohort of subjects who were diagnosed with the same psychiatric conditions; and third, to explore the potential roles of gender and age as effect modifiers. No association was found.

##### Monoamine Oxidase A

Monoamine oxidase A (MAOA), a mitochondrial outer membrane enzyme, is known to cause neurotransmitter degradation, including that of dopamine, norepinephrine, and serotonin. The promoter region of the MAOA gene, itself situated on chromosome Xp21-p11, is polymorphic in terms of how many copies of a 30-bp repeat it has. The alleles observed for this upstream variable number of tandem repeats (uVNTR) polymorphism include some with 3, 3.5, 4, and 5 repeats (3R, 3.5R, 4R, and 5R) (113). These uVNTR variants correspond to different transcriptional activities of the MAOA promoter, and these in turn give rise to varying expression levels of the MAOA gene.

A few recent studies have reported an association of the MAOA-uVNTR polymorphism with attempted suicide (114), while another study showed that there was a significantly higher frequency of the uVNTR two to three alleles in men who were violent suicide attempters than in those who were non-violent suicide attempters (115). We found more studies that did not show any association (42, 116). In the recent meta-analysis by Hung et al. (117), after pooling data on 1452 subjects with SB and 1198 unaffected controls, the authors failed to find any significant disparity in the allelic distribution of the MAOA-uVNTR polymorphism between subjects with SB and the male (OR = 0.85, 95% CI = 0.67–1.10, *p* = 0.22) or female controls (OR = 1.13, 95% CI = 0.94–1.36, *p* = 0.21). To better understand these divergent results, it is necessary to view them a new with a developmental approach. Indeed, in 2002, Caspi et al. (20) found that the MAOA-uVNTR polymorphism may reduce the risk of maltreated children growing up with increasingly antisocial behavior. Two other studies also find that SB might be influenced by the interaction between the MAOA-uVNTR polymorphism and trauma suffered in childhood (116). These various studies suggest that the MAOA-uVNTR genotype may not in itself exert an influence on SB but that it may interact with other environmental elements to produce the complex behavior observed.

#### Hypothalamic–Pituitary–Adrenal Axis

Studies designed to investigate the supposed causes of a defective regulation of HPA axis in SB have focused principally on two factors: (i) glucocorticoid receptor feedback mechanisms and (ii) the corticotrophin-release hormone (CRH) signaling system (13). Association studies have mainly focused on the CRHR1, CRHR2, FKBP5, CRHR, CRHBP, and NR3C1 genes. Wasserman et al. (118) studied two polymorphisms in CRHR1 (rs1396862 and rs4792887) and reported that the T-allele of rs4782887 conferred a risk of attempted suicide (*p* = 0.002). De Luca et al. (119) studied a cohort of 231 schizophrenia sufferers, 81 of whom had made a suicide attempt, and found CRHBP to be associated at significant levels with attempted suicide (*p* = 0.035).

In a family-based association study model, using 660 nuclear family trios and 519 healthy controls Ben-Efraim et al. (120) focused on the HPA axis and specifically on the role of the AVPR1B gene (arginine vasopressin receptor-1B) which was observed to associate with stress-related mood and anxiety disorders. In addition, they studied G × E interaction between AVPR1B variants and SLEs on any outcome. Their main results show a significant association (below the Bonferroni-corrected significance threshold of *p* = 0.0041) between variant rs33990840 and 6-SNP haplotypes with suicide attempt (SA) but predominantly concurrent with high depressive symptoms. On the other hand, genetic associations with lifetime diagnoses of depression and anxiety in SA or G × E interactions between AVPR1B variants and SLEs (childhood/adolescence/adulthood physical assault or sexual assault, and high lifetime SLEs) were not significant. An exploratory screen of interactions between AVPR1B and CRHR1 showed no support for gene–gene interactions on the studied outcomes.

#### Genes Involved in Neurotrophic Processes

Several lines of evidence from postmortem studies and expression studies indicate that brain-derived neurotrophic factor (BDNF) is a good candidate gene for involvement in SB (121). Further attention has been given to the Val66Met (rs6265) polymorphism in genetic studies of suicide. This particular polymorphism is an SNP in the BDNF gene, and it produces a valine (Val) to methionine (Met) substitution at codon 66 in the prodomain (BDNFMet).

We have listed a number of studies showing associations between Met allele and SBs in the context of various psychiatric diagnoses, including schizophrenia (122), bipolar disorder (123, 124), and depression (125, 126), and in several ethnic groups (Caucasian, Japanese, and Chinese). Table 3 summarizes the main selected studies.

**Table 3:** **Details of studies included in the review for neurotrophic factor gene**.

Reference	Variants	Suicide outcome/diagnosis	Sample investigated	Findings	Populations
**Brain-derived neurotrophic factor (BDNF) and other neurotrophic factors**					
Hong et al. (127)	Val66Met	SB-mood disorders	67 SB+ vs. 125 SB−	No association	Chinese
Hwang et al. (128)	Val66Met	SA-MDD (elderly inpatients)	22 SA+, 88 SA−, 171 controls	No association	Chinese
Iga et al. (125)	Val66Met	SA-MDD	23 SA+ vs. 131 SA−	Met66 allele carriers were more likely to have SB	Japanese
Huang and Lee (122)	Val66Met	SA-schizophrenia	16 SA+ vs. 116 SA−	Met/Met patient were more likely to have SB	Asian
Sarchiapone et al. (126)	Val66Met	SA-depressive disorders	97 SA+ vs. 73 SA−	A allele (AA + GA) * SA (65.3% vs. 50.5%; *p* = 0.05)	Caucasian (Slovenia)
Vincze et al. (124)	Val66Met + 3 SNPs	SA-bipolar disorders	176 SA+, 254 SA−, 370 controls	Val66 allele is risk allele for violent suicide attempt (*p* = 0.01)	Caucasian (France, Suisse)
Kim et al. (123)	Val66Met	SA-bipolar disorders	43SA+ vs. 126 SA−	Met/Met patient were more likely to have SB	Asian
Perroud et al. (129)	Val66Met	SA-psychiatric patients	615 SA+ non-violent, 198 SA+ violent	Val–Val genotype increase risk for violent attempt	Caucasian (France, Suisse)
Sears et al. (130)	Val66Met + 31 SNPs	SA-bipolar disorders	130 multiplex bipolar pedigrees, *n* = 795	7 SNPs of CCKBR* SA (*p* corr. < 0.05)	New Zealand
Zarrilli et al. (131)	Val66Met	Completed suicide	262 vs. 250 controls	No association	Caucasian (Slovenia)
Kohli et al. (132)	83 SNPs (NTRK2 and BDNF)	SA-depressive disorders	Discovery sample: 113 SA+, 366 SA−replication sample: 152 SA+, 592 SA−	rs11140714 (NTRK2) * SA *p* = 2.6 × 10^−4^ (*p* corr. = 0.043) and *p* corr. < 0.05 in replication sample. BDNF No association	African-American, German
Zouk et al. (66)	Val66Met + 3 SNPs	SA-bipolar disorders	74 SA+ vs. 86 SA−	rs4923463 (G/G) * violent SA (*p* = 0.03)	Brazilian
Pregelj et al. (133)	Val66Met	Completed suicide	359 vs. 201 controls	Met/Met and Met/Val genotypes are risk factors for violent completed suicide in female	Caucasian (Slovenia)
Strauss et al. (134)	10 SNPs (HOMER, NPTX)	SA-mood disorders	105 SA+ vs. 96 SA−	HOMER1 rs2290639* SA, NPTX2 rs705315, rs1681248 *SA (*p* corr. < 0.05)	African-American, European-American
Ropret et al. (135)	Val66Met + 7 SNPs	Completed suicide	486 vs. 289 controls	Haplotype C-A-T-C-C is risk haplotype for completed suicide	Caucasian (Slovenia)
Ratta-Apha et al. (136)	Val66Met + 6 SNPs	Completed suicide	307 vs. 380 healthy controls	No association	Various
Zai et al. (137)	Val66Met	SA-meta-analysis	8 studies: 433 cases, 1371 controls	OR Met-carrier = 1.25 (1.06–1.49) *p* = 0.008 OR Met = 1.22 (1.06–1.41) *p* = 0.006	Various
Clayden et al. (46)	Val66Met	SB-meta-analysis	7 studies: 1700 cases, 2548 controls	No association	Various
Ratta-Apha et al. (136)	Val66Met	CS-meta-analysis	3 studies: 921 cases, 825 controls	No association	Various
Ratta-Apha et al. (136)	Val66Met	CS-meta-analysis	6 studies: 471 cases, 967 controls	No association	Asian

*CS, completed suicide; MDD, major depressive disorder; SA, suicide attempt; SA+, suicide attempters; SA−, patient without a history of suicide attempt; SB, suicidal behavior; SI, suicide ideations; SNP, single-nucleotide polymorphism*.

*Associated with.

For example, a Slovenian team demonstrated the association between BDNF rs6265 polymorphism and suicide in a cohort of subjects with completed suicide (133). In the second part, this same team expanded their study by investigating several additional SNPs in the BDNF gene (rs7124442, rs10767664, rs962369, rs12273363, rs908867, rs1491850, and rs1491851). In this classic conception, the authors evaluated the differences in the allele, genotype, and haplotype frequency distributions of these seven SNPs between suicide completers and control subjects.

The control group was made up of subjects who died of natural causes or in road accidents. The authors were only able to demonstrate a statistical difference in haplotype analyses. The haplotype C–A–T–T–C was significantly associated with completed suicide. Additionally, single-marker analysis under four inheritance models with the adjustment for potential confounders, like age, gender, or alcohol dependence syndrome status also failed to reveal any associations. The diathesis–stress model is often used to include the biological and environmental contributions to SB, where the stressor is an environmental factor such as childhood abuse, substance abuse, or the experience of psychiatric illness. It is an approach which we have mentioned several times. The study by Perroud et al. (129) examined the interaction between BDNF rs6265 and a history of childhood sexual abuse and found that violent suicide attempts were associated with childhood sexual abuse only in those adults with a Val/Val genotype at rs6265. Sarchiapone et al. (126) found that the risk of a suicide attempt was significantly higher among depressed patients reporting higher levels of childhood emotional, physical, and sexual abuse.

More recently, Zouk et al. (66) found an association between genotype allele GA in rs4923463 and violent suicide attempt (*p* = 0.03) in patients with bipolar disorder. The other studied polymorphisms, including rs6265 (Val66Met), were not associated with any comorbidity. Further studies failed to report that Val66Met was associated with suicide attempt (131, 132). Zarrilli et al. (131) performed postmortem genotyping on 512 individuals, 262 of whom had committed suicide. There was no statistically significant difference between the level of the Met allele in the completed suicides compared to the controls. However, it should be noted that the authors do not specify the psychiatric characteristics of the cases and the controls. This may affect the results and influence the interpretation of the results.

Zai et al. (137) carried out the first meta-analysis of the functional BDNF marker Val66Met (rs6265, 196G > A) in SB, using data from 12 studies (total *n* = 3352 subjects, of whom 1202 had a history of suicide). This meta-analysis revealed a trend whereby the Met allele and Met-carrying genotypes conferred a risk of suicide (*p* = 0.032; ORMet = 1.16, 95% CI 1.01–1.32).

Working with a cohort de 130 multiplex bipolar pedigrees (*n* = 795), Sears et al. (130) found seven SNPs of CCKBR (rs2941025, rs2929183, rs2941023, rs2947025, rs2941029, rs2947029, and rs2947028) which were associated with suicide attempt and which remain significant after correction for multiple testing. No variant of the BDNF gene reaches the amended significance threshold (*p* ≤ 0.00156). This result is in contradiction with other case–control type studies and with the meta-analysis by Zai et al. (137) which were described earlier, in the BDNF section. The study by Sears et al. (130) has the advantage of having studied several BDNF polymorphisms, whereas the majority of other studies only tested the Val66Met SNP. The “cases” are all from a cohort of patients with bipolar disorder, which poses the question as to whether the significant CCKBR polymorphisms might be associated with bipolar disorder and not with suicide attempts. The authors point out that the SNPs tested had been previously studied in another cohort of bipolar patients and that no association with bipolar disorder had been found.

Recently, Ratta-Apha et al. (136) performed a meta-analysis that included six studies using Asian subjects (122, 123, 125, 127, 128, 136). The results demonstrated that the Met-allele had a tendency to be associated with the risk of suicide attempt (number of Met-alleles = 437: total number = 1.428, pool OR = 1.37, 95% CI = 1.01–1.86, *Z*-value = 2.047, *p* = 0.041). However, a meta-analysis that included three studies that used completed suicide as subjects (131, 133, 136) failed to show an association of the Met-allele with a risk of completed suicide (number of Met-alleles in completed suicide = 515: total number = 1.746, pool OR = 1.03, 95% CI = 0.88–1.19, *Z*-value = 0.315, *p* = 0.753).

With respect to the NTKR2 gene, Kohli et al. (132) showed an association of five tagging SNPs that are located within the NTRK2 locus with a lifetime history of SB within depressed patients in two independent German samples. On the other hand, the authors did not find any association with regard to BDNF. Perroud et al. (88) investigated the genetic predictors of an increase in suicidal ideation during the treatment of 796 adult subjects suffering from major depression who were treated with escitalopram or nortriptyline in Genome-based Therapeutic Drugs for Depression (GENDEP). The strongest association was detected for a SNP known as rs962369 in BDNF (*p* = 0.0015). In addition, a significant interaction was reported between the variants of BDNF and NTRK2 (*p* = 0.0003).

Strauss et al. (134) studied two genes that appear to be involved in neuroplasticity: HOMER1 and human neuronal pentraxin II (NPTX2). Their population is different from that of others because they focused on subjects who developed childhood-onset mood disorders (COMDs). This population is at high risk of SA and suicide. In their analyses, they made comparisons within the COMDs group and then compared the total COMD sample with healthy controls. After correction for multiple testing, none of the markers studied was significantly associated with COMDs. However, in the COMDs group, the authors report association between SA and HOMER1 rs2290639 genotype, as well as between SA and NPTX2 rs705315 and rs1681248 genotypes. The results should be interpreted with caution, given the stratification of the population. Indeed, the authors found more SA among African-Americans than in European-Americans. For example, rs2290639 is monomorphic for the A allele in HapMap YRI (African) sample, but not in the CEU (European) sample. As a result, the reduction in the risk of SA observed by the authors in heterozygous carriers of rs2290639 may be related to European-American ancestry.

#### Others Genes

In a population made up of 660 trios, Sokolowski et al. (138) reported several associations and linkage of SNPs in the GRIN2B and ODC1 genes with suicide attempt. In their haplotypic analysis of GRIN2B, the best significant results showed over-transmission for six haplotypes (all *p* < 4.0 × 10^−4^) of four to seven SNPs in length, all containing the minor risk C allele of SNP rs2268115. As in the study by Ben-Efraim et al. (74), the authors investigated G × E interactions, and especially physical and sexual abuse. In their analyses of G × E, the authors did not find significance for the GRIN2B or ODC1 SNPs which had shown direct genetic associations. However, one significant G × E was revealed between a third ODC1 SNP (rs7559979) and childhood/adolescent physical assault (*p* < 10^−4^).

Laje et al. (139) reported two other glutamatergic genes (GRIA3 and GRIK2) in relation to TESI. Similarly, a different study associated polyaminergic single-nucleotide polymorphisms explicitly with SBs (95).

In a sample of 77 trios (suicide attempters and both their parents), Wasserman et al. (140) investigated 250 genetic markers using TDT analysis. The authors showed that gene variants in the sodium channel, voltage gated, type VIII, alpha polypeptide (SCN8A) (*p* = 0.008), vesicle-associated membrane protein 4 (VAMP4) (*p* = 0.004), and prenylated Rab acceptor 1 (RABAC1) (*p* = 0.006) genes are over-transmitted in suicide attempt. In an independent replication sample comprised of 190 trios, the authors confirmed the data for the SCN8A (*p* = 0.005) and VAMP4 (*p* = 0.019) genes.

### Association Studies in Adolescent Population

Although SB is an important public health issue, few studies have been devoted to its genetic aspects. However, the hereditary component of SBs in adolescents has been well identified. The study by Brent et al. (5) is a reference in this field. This team studied the relatives of 58 adolescent suicide probands and 55 demographically similar controls. They clearly show that the rate of suicide attempts was increased in the first-degree relatives of suicide probands compared with the relatives of controls, even after adjusting for differences in rates of proband and familial Axis I and II disorders (odds ratio, 4.3; 95% confidence intervals, 1.1–16.6). We identified only very few studies devoted to the adolescent population. One possible reason for this is the difficulty of obtaining genetic research consents for minors. Two teams in the world have published several articles, the Zalsman et al. (34, 68, 72) and the Brent et al. (15) teams.

The oldest study we identified goes back to 2001 (34). Given the small number of studies, we have deliberately chosen to include it in our review. Zalsman et al. (34) attempted to clarify the role of the A218C polymorphism in intron 7 of the TPH gene. The family-based method, among others, was used, so as to limit the difficulty of sampling for the control group, in a cohort of 88 teenagers (of Ashkenazi Jewish origin) hospitalized for a suicide attempt. Allele frequencies were calculated and tested for association to phenotype using the HRR and TDT methods. The authors show that there was no significant allelic association of A218C polymorphism with suicide attempt or other phenotypic measures according to the HRR method (chi-square = 0.094; *p* = 0.76) and the TDT method (chi-square = 0.258; *p* = 0.61). In the same population, Zalsman et al. (68) tested T102C polymorphism (5-HTR2A gene) without demonstrating any significant association in allelic distribution between transmitted and non-transmitted alleles. Similarly, there was no significant association of genotype with any of the clinical traits. The same team conducted a case–control association study (72) in four groups of adolescents: (i) suicidal psychiatric inpatient adolescents (*N* = 35), (ii) non-suicidal psychiatric inpatient adolescents (*N* = 30), (iii) adolescents admitted to psychiatric emergency rooms due to a suicide attempt (*N* = 51), and (iv) a community-based control group (*N* = 95). The authors found that homozygosity for T (TT) of the HTR2A 102T/C polymorphism is associated with lower impulsivity (*p* = 0.03) and aggression (*p* = 0.01) compared with TC carriers and that a low activity MAOA was significantly associated with suicidality (*p* = 0.04). However, their analyses found no significant association between alleles of 5HTTLPR gene and suicidality.

Studying a subsample of adolescent depression (*n* = 155) sufferers who participated in the Treatment of SSRI-Resistant Depression in Adolescents (TORDIA) trial, Brent et al. (15) found that two polymorphisms in FKBP5 (rs1360780TT and rs3800373GG) were linked to suicide events (*n* = 18), even when they controlled for related covariates and treatment effects. Even though the number of suicidal events remains low, the authors correctly described the sample, the suicidal events were well characterized, and the effects of the treatment were taken into consideration in the analysis. In addition, this study is interesting from a pathophysiological point of view because the FKBP5 gene codes for a protein that decreases the sensitivity of the glucocorticoid receptor to the effect of corticosteroids. This result is consistent with previous studies linking SB with the insensitivity of the HPA axis to feedback and increased secretion of cortisol (94).

In summary, despite some interesting leads, the results all point to the same conclusions as in the adult population: no specific locus significantly associated with SBs has been identified so far, even when family-based methods are used.

### Genome-Wide Association Study

The use of association for the fine-mapping of candidate regions from linkage studies quickly gave way to more general or GWAS. One of the greatest benefits of GWAS is that it is “agnostic” or based on no prior assumptions. Usually, a simple regression analysis is used to systematically test each biallelic SNP across the genome for association with a trait or disease. Many generations of recombination create smaller regions of LD, which (with dense enough marker coverage) provides a substantially higher resolution than linkage and the potential to tag common causal variants (141). Researchers must make sure that the associated loci are not spurious associations which are due, for example, to population substructure or admixture (142). The use of hundreds of thousands of markers also necessitates very strict significance criteria, which makes it difficult to detect all but the largest effects. Li et al. (143) suggested that a *p*-value threshold of ~10^−7^ should be used as the genome-wide significance criterion for the earlier commercially available genotyping arrays, but marginally more rigorous *p*-value thresholds ~5 × 10^−8^ for more recent or merged commercially available genotyping arrays, ~10^−8^ for all the most frequent SNPs in the 1000 Genomes Project dataset and ~5 × 10^−8^ for the common SNPs only within genes.

We identified 11 GWAS. Table 4 summarizes the main results. Among these studies, we found three studies that focused on treatment emergent or treatment worsening suicidal ideation in patients who were taking antidepressant drugs (88–90) and eight GWAS (144–151) that were focused on suicidality in behavioral domains (suicide attempts or completed suicides).

**Table 4:** **Details of studies included in the review for genome-wide association study**.

Reference	Nb of SNPs	Sample investigated	Suicide outcome/diagnosis	Findings
Laje et al. (90)	109,365	*n* = 180, 90 cases	TESI: MDD	rs11628713** (PAPLN)
				rs10903034* (IL28RA)
Perroud et al. (88)	539,199	*n* = 706, 244 cases	TESI: MDD	rs11143230* (GDA)
				rs358592* (KCNIP4)
				rs4732812* (ELP3)
Menke et al. (89)	371,335	*n* = 397,32 cases	TESI: bipolar disorder	rs1037448* (TMEM138)
				rs10997044* (CTNNA3)
				rs1109089* (RHEB)
Perlis et al. (144)	1.9 × 10^−9^	*n* = 8737, 2805 cases	SA: MDD	MDD: rs2576377*** (ABI3BP)
			SA: bipolar disorder (BD)	BD-MDD: rs4918918* (SORBS1)
			SA: MDD or BD	BD-MDD: rs10854398* (B3GALT5)
				BD or MDD: rs1360550* (PRKCE)
Schosser et al. (146)	532,774	*n* = 2023, 251 cases	SA: MDD	rs4751955* (GFRA1) (Suicidality score)
				rs203136* (KIAA1244) (SA)
Willour et al. (145)	730,000	*n* = 5815, 2496 cases	SA/MDD	rs300774*** (2p25; ACP1, SH3YL1, FAM150B)
Galfalvy et al. (147)	37,344	*n* = 99,68 cases	Completed suicide	58* SNPs (19* genes)
Mullins et al. (148)	532,774	*n* = 3270, 426 cases	SA: mood disorders	No association
Galfalvy et al. (149)	1,014,770	*n* = 1810, 577 cases	CS and SA: psychiatric patients	15 SNPs* (STK3, ADAMTS14, PSME2, TBX20)
Zai et al. (150)	~1 million	*n* = 959 cases	SB (suicide severity scale): BD	rs10448042*, rs10448044* (IL7)
				rs3851150*, rs7244261* (TMX3)
Sokolowski et al. (151)	~1 million	660 nuclear family trios	SA-psychiatric patients	SNP-by-SNP GWAS: no association
				Polygenic associations: 750
				Neurodevelopmental genes (*p* corr. < 0.05)

*CS, completed suicide; MDD, major depressive disorder; SA, suicide attempt, SA+, suicide attempters; SB, suicidal behavior; SI, suicide ideations; SNP, single-nucleotide polymorphism*.

*Suggestive association p < 10^−5^.

**Genome-wide significant alpha 0.05 by experiment-wide correction.

***p < 5 × 10^−8^, genome-wide significant at alpha 0.05.

Laje et al. (90) followed up with the first GWAS in patients from the STAR*D clinical trial using the Illumina Human-1 BeadChip that samples 109,365 SNPs. One SNP (in PAPLN) was genome-wide significant (corrected *p* = 0.01, odds ratio 4.9), and another SNP (in IL28RA) was reported as suggestive.

Perroud et al. (88) published a GWAS of suicidality associated with treatment in the GENDEP 706-strong sample of patients being treated for major depression with either nortriptyline or escitalopram. The genetic marker that was found to have the most significant association with an increased level of suicidality (8.28 × 10^−7^) proved to be a single-nucleotide polymorphism (rs11143230) that was located 30 kb downstream of a gene that encoded guanine deaminase (GDA) on chromosome 9q21.13. In addition, treatment-specific SNPs (rs358592 in KCNIP4, rs4732812 near ELP3), drug × treatment interaction SNPs (rs1368607, rs2707159 in APOO gene, rs284668 near p53AIP1/RICS genes) together with a number of genes (in NTRK2, CCK, YWHAE, SCN8A, and CRHR2) from an additional candidate–gene analysis were all reported as suggestive associations. Menke et al. (89) carried out a GWAS on TESI, over the initial 12 weeks following treatment with various SSRIs in GSK-Munich and Munich Antidepressant Response Signature (MARS) cohorts. The discovery sample failed to show any association at significance level, whereby 79 suggestive SNP associations with the lowest *p*-values were instead used for testing in an independent replication sample. Following this two-phase analysis, 14 SNPs were reported to be suggestive, of which 6 SNPs were in high LD. These could be annotated to five distinct genes (TMEM138, CTNNA3, RHEB, CYBASC3, and AIMI), and the other three SNPs had an intergenic location. One SNP in GDA [a gene proposed in the Perroud et al. GWAS (88)] had a suggestive association with TESI, together with other candidate genes for neuro-psychiatric disorders (FKBP5, ABCB1).

In 2010, Perlis et al. (144) tested almost 2 million common genetic variants for association with a history of suicide attempts among 5815 individuals with bipolar disorder and 2922 individuals with major depression. In the bipolar cohort, they found five loci that included SNPs with a *p* value of <1 × 10^−5^, but none of them had a nominal *p* value of <0.05 in the cohort of bipolar disorder replication subjects. Among 1273 subjects with major depression, 6 loci had SNPs with a *p* value of <1 × 10^−5^; the minimum *p* value was 2.55 × 10^−8^ (rs2576377 in gene ABI3BP). But none of these regions had a *p* value of <0.05 in a second depression cohort. Furthermore, the authors examined association results in 19 genes that were previously suggested to be associated with suicide attempts. The two genes FKBP5 and NGFR (p75NTR) offered nominal evidence of association in bipolar disorder patients (uncorrected, *p* < 0.05) but which failed to persist after correction for 19 comparisons.

Schosser et al. (146) did not find any evidence for significant association at genome-wide level, and the strongest results in their study were not replicated in analysis of independent MDD cohorts with a similar assessment of SB. Their analysis of the candidate gene yielded some evidence of a polymorphism (rs10868235) in NTRK2 which had already been found to be associated with suicidality.

Willour et al. (145) reported a GWAS that compared SNP genotypes of 1201 bipolar (BP) suicide attempters with the genotypes of 1497 BP patients with no history of attempted suicide. After correction for multiple testing, none of the results from the replication sample had any significant association. On the other hand, an integrated meta-analysis investigation of both types of sample (discovery and replication) produced a significant genome-wide association for SNP rs300774.

The marker with the most significant association was rs300774 in an intergenic region at chromosomal region 2p25 containing the SH3YL1, ACP1, and FAM150B genes.

Galfalvy et al. (147) conducted a first study on completed suicides; sudden non-suicide-related deaths were used as controls. Their pilot study, which sought to identify candidate–gene regions that were associated with susceptibility to suicide, independently of the associated psychiatric diagnosis, found 22 SNPs in 19 genes using an SNP chip. Importantly, the majority of these genes have never been studied previously, and for many, their functions are unknown.

Mullins et al. (148) carried out GWAS and made the first application of polygenic score analysis to four patient cohorts with mood disorders, in an attempt to identify common genetic variants for mood disorders and SB. They used SNPs from three GWAS discovery studies of attempted suicides (BACCs, GSK-Munich, RADIANT), from one study of suicidal ideation (GENDEP) and from three studies of psychiatric disorders (PGC-MDD, PGC-BIP, and PGC-SCZ), in order to calculate polygenic scores for individual subjects in four validation datasets. They failed to detect any significant association evidence for any SNP in the GWAS or meta-analysis.

Galfalvy et al. (149) conducted a GWAS on completed suicides and patients having a history of attempted suicide with non-fatal outcome (*n* = 577) compared with psychiatric control and healthy volunteer groups (*n* = 1233). They used logistic regression to test association with genotype–SB. No SNP attained genome-wide significance in their study, but a number of SNPs in the ADAMTS14, STK3, TBX20, and PSME2 genes reached *p* < 1 × 10^−5^. They concluded that the most plausible candidate genes, ADAMTS14, PSME2 (both of them associated with inflammatory response), and TBX20 (brainstem motor neuron development), had not before been identified as being associated with SB.

Zai et al. (150) conducted a GWAS study of the severity of SB (from suicidal ideation to serious suicide attempt) using three independent BD samples and failed to detect significant genome-wide association of any of the markers tested in any of the BD samples. They identified markers in two chromosomal areas that were suggestively associated with suicide severity in bipolar patients (chromosome 8q12, near the proenkephalin gene PENK). The second stretch is located at chromosomal position 10p11.2, which contains the genes coiled-coil domain that contains 7 (CCDC7). As well as conducting GWAS of suicide severity, the authors carried out a GWAS of attempted suicides in three BD samples. They identified two regions of suggestive associations. The first region was localized to 8q12-q21 and was ~400 kb upstream of the interleukin 7 (IL7) gene. The second region was ~150 kb downstream of the thioredoxin-related transmembrane protein 3 coding TMX3 gene in 18q22.

Finally, the study by Sokolowski et al. (151) attempted to take account of the shortcomings of the earlier studies. With the major problem of GWAS being their low statistical power, the authors used the polygenic risk score (PRS) developed by Purcell et al. (152) PRS can show strong associations for many SNPs with small effects, and, in some cases, with small samples. PRS has also been used by Mulins et al. (148) to provide data concerning genetic overlap between different psychopathologies. To increase power, the authors used an *a priori* assumption with different genomic SNP subsets. The cases of SA were well identified from a cohort of 660 trios (nuclear family trios – all complete with both biological parents and one SA offspring per trio). The authors thus conducted the first-ever family-based GWAS. First, they performed a traditional SNP-by-SNP-based GWAS and they failed to reveal any significant genome-wide associations with SA. Similarly, when they focused on the 10 genes suggested in three previous GWAS on SB for follow-up (ABI3BP, B3GALT5, PRKCE, SORBS1, ACP1, KIAA1549L, LRRTM4, TMEM132C, GFRA1, and KIAA1244), no significant association was identified.

Second, the authors conducted PRS tests with several sources from the Psychiatric Genomics Consortium, and a PRS that was discovered and validated in the Genetic Investigation of Suicide and SA (GISS) revealed the polygenic association of SNPs in 750 neurodevelopmental genes, which was driven by the SA phenotype, rather than the major psychiatric diagnoses. Several results are worth noting: (i) the authors found evidence for polygenic associations of SNPs in neurodevelopmental genes in the SA subjects (even in the absence of major psychiatric diagnoses). (ii) The SCZ polygenes showed overlap with SA, and the degree of overlap depended on the presence or absence of diagnoses. (iii) The extended major histocompatibility complex region did not contribute to the overlap, but the authors delineated the genetic overlap to neurodevelopmental genes that partially overlapped with those identified by the GISS PRS. Among the 590 SA polygenes implicated here, there were several developmentally important functions and 16 of the SA polygenes have previously been studied in SB [BDNF, CDH10, CDH12, CDH13, CDH9, CREB1, DLK1, DLK2, EFEMP1, FOXN3, IL2, LSAMP, NCAM1, nerve growth factor (NGF), NTRK2, and TBC1D1]. These results, at genome-wide level, emphasize the importance of a polygenic neurodevelopmental etiology in SB. This is true not only for SBs but also for some other psychiatric disorders, especially in children and adolescents, for whom a developmental and integrative approach is essential.

## Discussion and Considerations for Future Directions

### Summary of the Current Review

Over the last decade, many teams from around the world have attempted to identify associations between genetic markers and SBs. It is recognized by all that single genes might not explain the full risk of developing SBs. In summary, we have identified several studies that have shown an association of genetic polymorphisms with SBs, in line with previous reviews (31–33). The strongest results from meta-analyses support the combination of SB with variants in TPH1-rs1800532 (43, 46, 84), SLC6A4-5-HTTLPR- (46, 84), COMT-rs4680-(67) or BDNF-rs6265 (137).

Results to date from GWAS are unsatisfactory, with most studies showing no evidence of association at genome-wide significant level (89, 145, 147–149) or only marginally (90, 146, 150). Studies which did show an association (*p* < 5 × 10^−8^) (88, 144) failed to replicate the results.

Several pathways have been mentioned in an attempt to understand the lack of reproducibility and disappointing results. Consequently, we shall now review and discuss the following: (i) sample characteristics and confounding factors; (ii) statistical limits; (iii) gene–gene interactions; (iv) gene, environment, and by time interactions; and (v) technological and theoretical limits.

### Sample Characteristics and Confounding Factors

The ability to identify significant associations and the relevance of such information to suicidality is linked both to the number of subjects in each group and to the method used to define the groups. Although the family transmission of suicidality would tend to suggest that suicide is a separate phenomenon from psychopathology (10), there is need to carefully control biological factors that are associated with psychopathology, and this is a process which poses significant methodological and operational challenges. Several diagnoses frequently associated with suicide like, for example, bipolar disorder, major depression, schizophrenia, or alcoholism, have been routinely included in studies curried out thus far, and use has been made of various methods to distinguish their effects from those linked to suicide. In contrast, axis II has rarely been considered, and it appears to be a confusing factor, considering the importance of personality traits such as impulsivity/aggressiveness.

Turecki (18) suggested that the studies having the best chance of properly controlling psychopathology were those which, within psychopathological groups, compared subjects who committed suicide with those whose death was due to another cause. However, it poses a considerable operational challenge to constitute a control group of subjects suffering from psychopathology which is comparable to the suicide group with respect to a number of other variables.

Second, variations in the definition of SB in the studies on genetic association are considerable, a fact which would tend to render comparisons between the results obtained somewhat rash. The definition of suicidal ideation includes suicide threats or thoughts that produce no action, and the precise clinical definition of the concept is still inadequate and confusing. A number of studies offer evidence showing that suicidal ideation is very different, in terms of phenotype, from suicide attempt. An excess level of suicidal ideation was observed in the family environment of suicide victims, but it was not found to be significant once adjustments were made to take account of psychiatric disorders (5). The suggestion has been made that ideation might cosegregate with psychiatric disorders, while the tendency to move from ideation to action is, at least to some extent, the result of a different genetic diathesis (91). The logical conclusion would seem to be that molecular genetic studies on attempted suicide cases should not seek to address the concept of suicidal ideation.

Some have proposed the use of eminently heritable phenotypes in genetic analyses as being the most promising way of identifying real genetic associations. A strategy of heritable intermediate phenotypes (endophenotypes) has been proposed by the American Foundation for Suicide Prevention (91). A number of promising endophenotypes that have been put forward for genetic studies on suicide include traits of aggression/impulsivity. However, the choice of such suicidality-related phenotypes is not a light matter.

### Statistical Limits

In the genetics of complex disease, it is necessary to limit both type I and type II errors. The value of studies concerned with allelic association is mainly a function of the size of the samples, the effect size of the susceptibility loci, how strong the linkage disequilibrium with a marker is, and how frequently susceptibility and marker alleles occur. Multiple testing, the rates of false positives and statistical significance levels are important issues in genetic association studies. Several statistical techniques are being developed for multiple comparison correction, but the ability to replicate findings of genetic associations in unrelated population samples is still the ultimate benchmark for complex disease genetics (153). GWAS picks up on alleles in the population which occur commonly, but which are each unlikely to have more than a very slight incidence on what is a complex phenotype (154).

Simultaneously investigating thousands of SNPs, which individually do not reach significance, could explain a greater amount of the heritability. To increase the power to identify disease variants, several genetic markers could be studied simultaneously. The cumulative genetic effect of multiple SNPs is more likely to have a higher heritability than any of the individual SNPs. Other methods have been proposed, including global haplotype tests, regression methods, and multimarker tests; these tests address multiple testing by combining multiple SNPs into a single test (155). Polygenic score analysis has recently generated much interest for assessing the explanatory power of an ensemble of markers. A GWAS is conducted on an initial training sample, and the markers are ranked by their evidence for association, usually their *p*-values. An independent replication sample is then analyzed by constructing, for each subject, a polygenic score consisting of the weighted sum of its trait-associated alleles, for some subset of top ranking markers (156). This approach has been used by the Psychiatric Genomics Consortium and the International Schizophrenia Consortium to investigate major depression and schizophrenia (157). This recent method could explain a larger component of the heritability of SB than would individual alleles that each has small effect sizes.

### Gene, Environment, and by Time Interactions

Gene–environment interactions might to some extent account for variations in the link between the experience of stressful events and the emergence and severity of a given major depression and SB episode in young subjects. For example, the well-known study by Caspi et al. (20) showed that depression accompanied by suicidal ideation or attempted suicide is to be predicted in children, adolescents, and young adults who are carriers of the S allele of the 5-HTTLPR polymorphism. This pattern involves a complex area of research because it is difficult to understand the impact of the environment. However, several studies have shown interesting results, mainly those who have considered adverse life events (19). Moreover, age appears to also be a factor that has not sufficiently been taken into consideration. Studies that used MRI and fMRI showed both alterations related to age and differences related to gender in gray and white matter over the period of adolescence (158). These results could go some way to explaining why a large number of psychiatric disorders, including SB, manifest themselves during this period of life and might explain the gender-related differences seen in adolescent SB, namely that females have a greater tendency to attempt suicide than males, who tend to achieve completed suicide more frequently. Zalsman et al. (72) suggested that the fact of restricting the investigation of SB in adolescents simply to the interaction between gene and environment might prevent researchers from detecting other complex interaction factors, which involve timing. It seems legitimate to speculate that it is only when particular genotypes are exposed to specific environment-related risks during a critical period of brain development that suicidality would be the outcome.

### Gene–Gene Interactions

A reason that is frequently given to explain why genetic studies of complex diseases have met with such scant success is the interactions which have been observed to exist between loci. Given that there is recognition of the complexity of the genetic architecture, with several levels of interaction, the fear is that the effect will be missed if one examines it in isolation, without taking into account the possible interactions it may have with the other factors. Various methods and software packages have therefore been designed to take account of statistical interactions between loci in the analysis of data provided by studies of genetic association. In a large review, Cordell (159) offers a critical survey of the methodological approaches (with the associated software) which are in current use for the detection of interactions between genetic loci identified as contributing to genetic disease in humans. She concludes that, even though the exact details of the methodologies may differ, there are, in many instances, tight conceptual links between the various approaches, and a correct apprehension of such links can perhaps best be obtained through an improved understanding of the difference between testing for interactions as against testing for associations while taking account of interactions. In her review, few studies have taken into consideration the gene–gene interactions. In our study, we include several studies which analyzed the gene–gene interactions. For example, De Luca et al. (119), studying a cohort of 231 patients suffering from schizophrenia, found a supposedly significant interaction between CRHBP rs1875999 and CRHR1 rs16940665 as regards the seriousness of SB, and more recently, Perroud et al. (88), in their recent GENDEP clinical trial sample, reported NTRK2 and BDNF polymorphisms to interact significantly in suicidal ideation.

### Technological and Theoretical Limits

In our review, all of the studies of association are based on the hypothesis of the “common disease common variant” (CDCV). According to the CDCV hypothesis, common diseases are triggered by common variants, with effects that range from small to modest. According to the alternative theory, the “common disease rare variant” (CDRV) hypothesis, there is an extremely high level of allelic heterogeneity for complex traits, with disease etiology being the collective result of numerous rare variants having moderate to high penetrances.

In terms of its practical implications, the issue may be split up into two parts. Should the CDCV hypothesis be valid, then the application of the paradigm of positional cloning to the task of mapping disease genes would be considerably facilitated since a common allele would be more easily located. If, on the other hand, common diseases are caused by rare variants, then the task of identifying such genetic susceptibility variants would represent a real challenge. Although there exist a substantial body of evidence to suggest that the CDCV and CDRV theories are both valid, a model for complex traits which would come closer to reality is likely to be that functional variants occur over a wide range of allele frequencies from rare to common, even for the same susceptibility gene (160). The technological progress made recently in high-throughput sequencing platforms should shortly enable the extension of association studies to rarely occurring and very uncommon variants, especially in targeted exon resequencing. They predict that uncommon variants are likely to be enriched for functional alleles and to display larger effect sizes than do common variants, in accordance with the hypothesis that functional allelic variants are subjected to the pressure of purifying selection.

For identifying rare variants, exome sequencing in families is more effective than case–control studies. Given that disease alleles are shared among affected family members through identity-by-descent, the number of alleles needing to be considered could be limited by segregation analysis. However, this method is more difficult with suicide, which is a rare event.

To identify relevant disease-related genes, instead of investigating SNPs, another approach is to carry out genome-wide exploration to determine gains and losses of copy numbers. These copy number variants (CNVs) have no greater intrinsic pathogenic potential than a single-nucleotide change, but their size means that they can potentially raise or reduce the gene product at each CNV intersecting a gene or to modify the genomic environment with *cis* or *trans* effects which are potentially far-reaching. Based on single-nucleotide polymorphism array data, Gross et al. (161) followed the Penn-CNV standards to detect CNVs in 1608 cases (suicide and suicide attempt) and 1133 controls. Although the initial algorithms determined the presence of CNVs on chromosomes 6 and 12 in seven and eight cases respectively, compared with none of the controls, visual inspection of the raw data did not support this finding. But the authors were unable to confirm these results by CNV-specific real-time polymerase chain reaction. Additionally, they did not find any association between the frequency or length of rare CNVs and SBs.

Using subjects who were collected as part of STAR*D, Perlis et al. (162) genotyped 189 patients suffering from MD who had a history of attempted suicide, and 1073 subjects suffering from MDD but who had not previously attempted suicide. The implication of their results is that no distinction can be made between these two groups in terms of a given CNV, and that should a given CNV be associated with attempted suicide in MD, it would probably be a common one. In other words, they failed to identify any CNVs that were not reported in the Database of Genomic Variants, which suggests that suicide attempt status is not influenced by any copy changes in the STAR*D sample. According to the authors, to be able to observe any effect of a common CNV, it would be necessary to increase sample sizes >20- to 30-fold. Statistical limits are also an obstacle. For example, there is a technological limit because the level of detection of the size of a CNV is also a limit. Despite technological advances for association studies, only a comparatively small part of the heritability of the majority of complex traits has received an explanation and the variants which the studies concerned have identified are characterized by small effect sizes. This circumstance has raised the major, controversial, question of where the “missing heritability” of complex disorders is hiding (163). According to Nadeau et al. (163), perhaps the role of heritability has been exaggerated. Another possibility is that the missing variants might be located in areas of the genome which have as yet been insufficiently explored or in classes of genetic variation which have as yet been insufficiently tested. Alternatively, perhaps the genetic variants are going undetected because of their rarity and the smallness of their effects. Or maybe the complexity of genetic mechanisms has been underestimated, in the sense that very numerous closely related genes may display effects which depend on context and are non-additive.

Further to that, Nadeau et al. (163) raises the question of transgenerational genetic effects, whereby phenotype variations in the current generation may very likely result from genetic variants in preceding generations.

The more recent contribution made by epigenetic studies would appear to be an interesting path for understanding SBs. Epigenetic mechanisms, such as DNA methylation and chromatin restructuring, can be altered by environmental factors but the complexity of the epigenome is not fully understood (164). The recent review by Turecki et al. (165) assesses emerging data for the role of epigenetic mechanisms in stress-related psychiatric disorders. The Turecki team has published numerous studies on epigenetic mechanisms that are possibly related to SBs. They have reported promoter-wide hypermethylation of the ribosomal RNA gene promoter (166), hypermethylation of the tropomyosin-related kinase B (167), hypermethylation in the promoter of the glucocorticoid receptor (168), or hypermethylation in the promoter of spermine oxidase (169), in the brain of suicide subjects. We may also cite the studies on BDNF promoter hypermethylation (170) or on SKA2 DNA methylation (171).

In conclusion, although the studies on the heritability of SB have shown a strong genetic component, genetic association studies have failed to clearly identify specific markers contributing to this genetic liability. Numerous genes appear to be involved in the emergence of SBs. Several neurobiological pathways are involved, with multiple interplay of genetic and environmental factors. While the complexity is daunting, advances in statistical and genetic methodologies as well as increasingly informative developmental studies can help sustain an approach of guarded optimism. A better understanding of the genes that are involved in SB and their interaction with genetic and non-genetic factors could help in the development of more effective screening, prevention, and management of SB.

## Author Contributions

All authors contributed to the design of the study. BM participated in the design of the study and drafted the manuscript. PG participated in the design of the study, collected the data, and helped to draft the manuscript. DC helped to draft the manuscript. CL conceived the study, participated in its design, and drafted the manuscript. TF helped for discussion. All authors have reviewed and approved the manuscript.

## Conflict of Interest Statement

The authors declare that the research was conducted in the absence of any commercial or financial relationships that could be construed as a potential conflict of interest.
